# Time-dependent tuning of balance control and aftereffects following optical flow perturbation training in older adults

**DOI:** 10.1186/s12984-019-0555-3

**Published:** 2019-07-01

**Authors:** Jackson T. Richards, Brian P. Selgrade, Mu Qiao, Prudence Plummer, Erik A. Wikstrom, Jason R. Franz

**Affiliations:** 1 Joint Department of Biomedical Engineering, University of North Carolina at Chapel Hill and North Carolina State University, 10206C Mary Ellen Jones Building, CB 7575, Chapel Hill, NC 27599 USA; 2 Department of Kinesiology, Louisiana Tech University, Ruston, LA USA; 3 Division of Physical Therapy, University of North Carolina at Chapel Hill, Chapel Hill, NC USA; 4 Department of Exercise and Sport Science, University of North Carolina at Chapel Hill, Chapel Hill, NC USA

**Keywords:** Walking, Virtual reality, Elderly, Stability, Coactivation

## Abstract

**Background:**

Walking balance in older adults is disproportionately susceptible to lateral instability provoked by optical flow perturbations. The prolonged exposure to these perturbations could promote reactive balance control and increased balance confidence in older adults, but this scientific premise has yet to be investigated. This proof of concept study was designed to investigate the propensity for time-dependent tuning of walking balance control and the presence of aftereffects in older adults following a single session of optical flow perturbation training.

**Methods:**

Thirteen older adults participated in a randomized, crossover design performed on different days that included 10 min of treadmill walking with (experimental session) and without (control session) optical flow perturbations. We used electromyographic recordings of leg muscle activity and 3D motion capture to quantify foot placement kinematics, lateral margin of stability, and antagonist coactivation during normal walking (baseline), early (min 1) and late (min 10) responses to perturbations, and aftereffects immediately following perturbation cessation (post).

**Results:**

At their onset, perturbations elicited 17% wider and 7% shorter steps, higher step width and length variability (+171% and +132%, respectively), larger and more variable margins of stability (MoS), and roughly twice the antagonist leg muscle coactivation (*p*-values<0.05). Despite continued perturbations, most outcomes returned to values observed during normal, unperturbed walking by the end of prolonged exposure. After 10 min of perturbation training and their subsequent cessation, older adults walked with longer and more narrow steps, modest increases in foot placement variability, and roughly half the MoS variability and antagonist lower leg muscle coactivation as they did before training.

**Conclusions:**

Findings suggest that older adults: (*i*) respond to the onset of perturbations using generalized anticipatory balance control, (*ii*) deprioritize that strategy following prolonged exposure to perturbations, and (*iii*) upon removal of perturbations, exhibit short-term aftereffects that indicate a lessening of anticipatory control, an increase in reactive control, and/or increased balance confidence. We consider this an early, proof-of-concept study into the clinical utility of prolonged exposure to optical flow perturbations as a training tool for corrective motor adjustments relevant to walking balance integrity toward reinforcing task-specific, reactive control and/or improving balance confidence in older adults.

**Trial registration:**

clinicaltrials.gov (NCT03341728). Registered 14 November 2017.

**Electronic supplementary material:**

The online version of this article (10.1186/s12984-019-0555-3) contains supplementary material, which is available to authorized users.

## Introduction

Older adults are at an exceptionally high risk of falls, and most of those falls occur during locomotor activities such as walking [1]. Moreover, 20–30% of these falls result in moderate to severe injury with enormous personal and financial costs [2]. Unfortunately, our society is facing a growing dilemma – evidence suggests that the rate of injurious falls is accelerating, with 2.4 million emergency department visits related to falls in 2011, up 46% from 2001 despite only a 17% increase in the older adult population [3]. Thus, despite increased awareness and significant scientific investment, there is a critical need for innovation in our development of novel and more effective techniques to improve walking balance and prevent falls in older adults.

Lateral balance in walking is achieved through coordinated adjustments between the continuous control of posture (i.e., head and trunk stabilization) and the discrete (step-to-step) control of foot placement (i.e., step width) [4–7]. Accordingly, walking balance integrity is routinely quantified using step-to-step variations in foot placement (e.g., step width variability [8, 9]) and/or the relation between foot placement and postural deviations (e.g., margin of stability [10]). Orchestrating those adjustments, particularly in response to balance challenges, depends on appropriate motor planning and execution, which in turn depend on having accurate and reliable sensory feedback. Unfortunately, compared to young adults, many older adults succumb to a disproportionate loss of proprioceptive acuity and tactile sensation that may compromise balance control [11, 12]. However, through an adaptive process of multi-sensory reweighting [13], older adults retain some capacity to compensate for distal sensory impairment, and do so in part by relying more on visual feedback for balance control – effects that are even more pronounced in older adults with a history of falls [14, 15].

By leveraging their reliance on visual feedback, optical flow perturbations designed to elicit the visual perception of instability routinely provoke corrective motor responses to preserve walking balance that are larger in older non-fallers than in young adults [8, 16] and larger still in older fallers [17, 18]. The cumulative insights from those studies suggests that older adults respond to those unpredictable balance challenges, at least during early exposure, by adopting what may be interpreted as a more cautious or generalized anticipatory balance control strategy. This logical response from participants at the onset of balance perturbations likely only reinforces a strategy normally adopted more by older than young adults during their everyday tasks. Indeed, compared to young adults, older adults choose to walk with wider and shorter steps and increased antagonist leg muscle coactivation [19]. The latter is a hallmark feature of elderly gait, and is associated at least in part to a fear of falling [20]. Indeed, antagonist coactivation is often considered a generalized anticipatory strategy to increase joint stiffness as a means to mitigate any effect of unexpected balance disturbances [21]. However, we posit that such generalized anticipatory control comes at the cost of inhibiting agonist muscle function and the ability to generate rapid, task-specific balance corrections.

Task-specific training may promote reactive balance control in older adults, which could improve balance integrity and mitigate falls risk. As one example, Grabiner et al. (2012) found that a task-specific training protocol with treadmill-delivered postural disturbances improved reactive control and reduced the occurrence of laboratory-induced trips compared to participants not receiving training [22]. Given older adults’ remarkable susceptibility to optical flow perturbations, which ultimately require significant and task-specific adjustments in lateral foot placement and posture to prevent falling, could a similar paradigm be used to increase balance confidence and promote reactive balance control during walking? In a recent proof-of-concept study, Thompson and Franz [23] studied young adults’ acute and time-dependent response to prolonged exposure to optical flow perturbations during treadmill walking. Those authors concluded that gait kinematics in young adults alluded to a shift from generalized anticipatory control at the onset of perturbations (i.e., shorter, wider steps) to a more reactive, task-specific strategy of step-to-step adjustments (i.e., more variable step length and width) as perturbations continued. Reinforcing reactive, task-specific balance control through the use of optical flow perturbation training may have clinical utility for individuals at risk of falls, particularly with the advent and widespread availability of wearable low-cost virtual reality technology. However, no study to date has examined the time-dependent response of older adults to prolonged exposure to optical flow perturbations.

Therefore, the purpose of this study was to investigate the propensity for time-dependent tuning of walking balance control and the presence of short-term aftereffects in older adults following prolonged exposure to the visual perception of gait instability. We used a virtual reality environment to prescribe continuous mediolateral optical flow balance perturbations during treadmill walking in a fully-randomized, crossover design that included a control session of prolonged, unperturbed walking. We first hypothesized that, at the onset of optical flow perturbations, older adults would adopt a generalized anticipatory balance control strategy, walking with shorter, wider steps, larger margins of stability, and greater antagonist leg muscle coactivation compared to walking normally. Second, we hypothesized that older adults would deprioritize that strategy following prolonged exposure to perturbations, such that those metrics of walking balance control would trend towards values seen during normal walking. Finally, we hypothesized that older adults would exhibit aftereffects following cessation of optical flow perturbations indicative of lessened anticipatory balance control, increased reactive balance control, and/or increased balance confidence. Specifically, we anticipated that these aftereffects would include longer and more narrow steps, smaller margins of stability, and less antagonist leg muscle coactivation in older adults compared to normal walking.

## Methods

Thirteen older adult participants (mean ± standard deviation; age: 78.7 ± 6.6 years, height: 1.67 ± 0.09 m, weight: 67.5 ± 11.3 kg, 9F/4M) participated in this experiment, which was part of a larger study. Participants completed a health questionnaire prior to participating to ensure that they were: free of known neurological, musculoskeletal, cardiovascular, ophthalmological, or orthopedic disorders and able to walk without an assistive aid (i.e., walker, cane). Four participants self-reported a prior history of one fall in the last year, but all participants were analyzed together. Prior to testing, all participants provided written informed consent according to the University of North Carolina Biomedical Sciences Institutional Review Board. To provide some context for the sensorimotor integrity of our older participants, we assessed bilateral plantar sensation using a Semmes-Weinstein monofilament test with a 4–2-1 design [20] (Table 1). We also reference young adult data from Thompson and Franz [23] from ten participants (age: 25.4 ± 3.8 years, height: 1.75 ± 0.10 m, weight: 74.4 ± 16.7 kg, 4F/6M).

**Table 1:** Individual subject characteristics

Subject ID	Age (years)	Sex	Height (m)	Mass (kg)	PWS (m/s)	Falls History	SW Heel	SW MTP
A	73	F	1.81	76.36	1.12	No	5.07	4.74
B	83	M	1.77	86.53	1.16	No	6.10	5.88
C	76	F	1.73	65.01	0.92	No	4.56	4.74
D	74	M	1.74	73.73	1.51	No	4.08	3.84
E	74	F	1.65	57.66	1.24	No	3.84	3.22
F	80	F	1.53	66.01	1.31	No	4.56	4.31
G	79	F	1.67	47.22	1.30	No	5.46	5.07
H	68	F	1.67	60.38	1.25	No	4.56	3.22
I	79	M	1.77	82.63	1.08	No	5.07	4.93
J	87	F	1.61	67.74	0.84	Yes	4.19	4.68
K	90	M	1.61	79.45	0.65	Yes	4.87	4.08
L	73	F	1.71	64.38	1.25	Yes	4.56	4.08
M	87	F	1.52	51.76	1.04	Yes	4.31	4.08

*PWS* preferred walking speed, *Falls history* 1 fall in the preceding year, *SW* semmes weinstin monoliment test

*MTP* metatarsophalangeal joint

### Experimental procedure

**Fig. 1:**
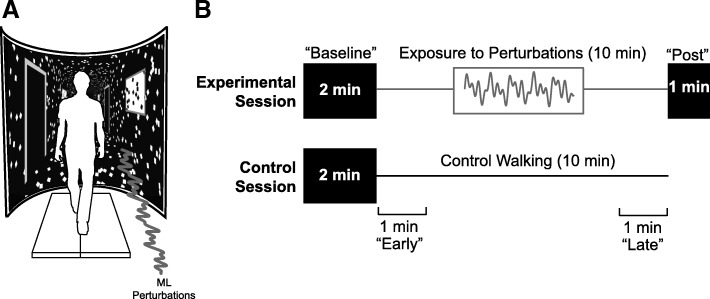
**a** Participants walked on a treadmill while watching a speed-matched immersive virtual hallway with and without continuous mediolateral (ML) optical flow perturbations with an amplitude of 0.35 m, applied as detailed in the current methods section. **b** Participants participated in two sessions performed on separate days separated by at least 1 week and in fully randomized order. Both sessions consisted of a 2 min “baseline” walking period without perturbations. In one session (“Experimental”), participants then walked for 10 min in the presence of perturbations, followed immediately by a 1-min “post” period without perturbations. In the other session (“Control”), participants simply walked for 10 min without perturbations. For analysis, we refer to the first and last minute of each 10-min prolonged walk as “early” and “late”, respectively

This study involved experimental testing on two separate days, separated by at least 1 week, and performed in a randomized order. Specifically, we reversed the session assignment with each subject determined by their enrollment date, and subjects were not explicitly informed of their session order. Participants started their first session walking down a 10-m walkway at their self-selected comfortable speed. From this, we calculated participants’ preferred overground walking speed as the average of three times taken to traverse the middle 4 m of the walkway (1.20 ± 0.25 m/s). Participants then walked on the treadmill for 5 min to acclimate and allow their movement patterns to stabilize. Some participants reported that the treadmill felt faster than they would prefer. In those cases, we slowed the treadmill speed by 10% for the remainder of the experiment, yielding a group-average tested speed of 1.13 ± 0.25 m/s. Participants then completed all treadmill walking trials on a force-sensing treadmill (Bertec, Inc., Columbus, OH) at their preferred speed while watching a custom, speed-matched virtual hallway (Fig. 1a, Additional file 1: VideoS1). The virtual hallway was controlled using a Simulink Desktop Real-Time™ model (Mathworks, Natick, MA) and was rear-projected onto a semicircular curved screen surrounding the treadmill that measured 2.90 m high and 2.54 m wide. Prior to completing the treadmill trials, we instructed participants simply to “walk normally while looking down the hallway” so that they could naturally respond to the prescribed changes in visual stimuli we described in more detail below.

Figure 1b summarizes our experimental protocol. In both sessions, participants completed a 2-min treadmill walking trial in which the virtual hallway was speed-matched but included no optical flow perturbations (i.e., “Baseline”). Here, optical flow provided by the black and white tiled walls provided the visual perception of walking straight down the hallway. In the experimental session, participants then completed an 11-min walking trial while being exposed to continuous optical flow perturbations during the first 10 min. Specifically, to the speed-matched virtual hallway, we added continuous mediolateral oscillations of optical flow which were prescribed as the sum of three sine waves (phase, φ = 0), such that a full 0.35 m amplitude was applied at 0.250 Hz and half that amplitude was applied at 0.125 Hz and 0.442 Hz. The dynamics of this particular mediolateral oscillation have been used extensively in prior studies, with well-documented effects on gait kinematics and leg muscle activities and in young and older adult cohorts [9, 17, 23–25]. Perturbations were consistent with the visual feedback associated with mediolateral translation of the head and trunk, such that the black rectangle representing the far end of the hallway remained stationary while the black and white tiled walls in the foreground of the hallway oscillated mediolaterally. Accordingly, the visual stimuli were designed to induce balance corrections rather than heading corrections. At the end of the 10th minute, optical flow perturbations ceased and participants continued walking without perturbations for 1 min. In the control session, we replaced the prolonged optical flow perturbation trial with a 10-min control walking trial without perturbations. Our rationale was to isolate time-dependent changes in elderly gait due to adaptation to optical flow perturbations from those due to prolonged treadmill walking alone. During treadmill walking, participants were asked not to hold onto the handrails at any time and wore an overhead support harness.

### Measurements

For the duration of each trial, a 14-camera motion capture system (Motion Analysis Corp., Santa Rosa, CA) operating at 100 Hz recorded the three-dimensional positions of markers placed on participants’ left and right heels, 1st and 5th metatarsophalangeal joints, and sacrum, with the latter acting as a surrogate for center of mass in the coronal plane [26]. For the session including optical flow perturbations, after shaving and cleaning the skin with alcohol, we placed wireless single differential electrodes (Trigno, Delsys, Inc., Boston, MA) over the following six right leg muscles: medial gastrocnemius (MG), soleus (SOL), tibialis anterior (TA), vastus lateralis (VL), medial hamstring (MH). We determined muscle placement according to the recommendations of Cram and Kasman [27], and recorded electromyographic (EMG) data at 1000 Hz. We visually inspected the EMG signals while participants contracted each instrumented muscle to ensure signal quality and electrode placement.

### Data analysis

We first derived foot placement outcome measures from our kinematic data. We filtered marker trajectories using a 4th order recursive Butterworth filter with a 4 Hz low-pass cutoff frequency. Because we sought to investigate natural adaption to perturbations over time, we did not instruct participants to keep one foot on each treadmill belt. We used these filtered marker trajectories and methods described by Zeni et al. [28] to identify the instants of right and left heel-strikes from the peak anterior heel positions relative to the sacral marker. Right and left toe-offs were calculated similarly as the peak posterior distance from the sacral marker to the corresponding 1st metatarsophalangeal joint. We calculated time series of step widths by averaging heel marker positions during midstance prior to heel rise (i.e., 12–25% of the gait cycle) and determining the mediolateral distance between consecutive steps. We computed step lengths using the relative anterior-posterior positions of successive heel markers at 20% of the gait cycle plus the treadmill belt translation during each step. To test for time-dependent changes in step width and step length over the two prolonged treadmill walking trials, rather than step to step variations, we used a moving average with a window of 30 footsteps to remove short term fluctuations [29–32]. Using their original time series, we also calculated step width and length variabilities (SWV and SLV, respectively) as the standard deviation of step widths and step lengths over steps occurring in non-overlapping, 60 s bins.

We then used our kinematic outcome measures to calculate margin of stability (MoS) using previously described methods [10, 33]. Specifically, we defined MoS as the difference in position between the lateral base of support (BoS) and the extrapolated center of mass (XCoM) as follows:

1 $$ \mathrm{XCoM}=\mathrm{x}+\frac{\dot{\mathrm{x}}}{\upomega_0} $$

2 $$ \mathrm{MoS}=\mathrm{XCoM}-\mathrm{BoS} $$

where ω_0_ is the natural frequency of an inverted pendulum model of the stance phase (ω_0_^2^ = *g*/*L*), g is acceleration due to gravity (9.81 m/s^2^), L is leg length, and x is the position of the sacrum marker, used here as a surrogate for the body’s center of mass. We calculated leg length as the distance from the sacral marker to the heel marker at heel-strike averaged across all strides bilaterally for each subject. We defined the lateral base of support as the lateral position of the 5th metatarsophalangeal marker [34]. Consistent with the work of Young and Dingwell [33], we first evaluated lateral margin of stability and its variability at the minimum during each stance phase given the potential for falls when margin of stability is its smallest. In addition, we include MoS at heel-strike based on evidence that (*i*) a loss of balance is relatively likely at heel-strike [35] and (ii) it can be challenging (i.e., require more muscle activity) to regain balance lost at heel-strike compared to other times during the gait cycle [36].

A custom MATLAB script processed all EMG data (Mathworks, Inc., Natick, MA). EMG signals were bandpass filtered (20–400 Hz), full-wave rectified, and normalized to the mean value over the final minute of the 2-min Baseline walking trial for that experimental session. Using the instants of right heel-strikes determined earlier, we computed average muscle activation profiles from which we calculated antagonist muscle coactivation using methods described by Falconer and Winter [37] as follows:

3 $$ \mathrm{Coactivation}=2\left({\mathrm{iEMG}}_{\mathrm{ant}}\right)/{\mathrm{iEMG}}_{\mathrm{tot}} $$

where iEMG_ant_ is integrated EMG of the less active (antagonist) muscle and iEMG_tot_ is the sum of both muscles’ integrated EMG values. We calculated coactivation for three pairs of muscles with opposing actions: (*i*) TA and SOL, (*ii*) TA and MG, and (*iii*) VL and MH. Specifically, we separately computed coactivation indices for each of these three muscle pairs during the stance.

### Statistical analysis

We evaluated the following dependent variables: step length, step width, step length variability, step width variability, stance and swing phase antagonist coactivation for three muscle pairs (VL-MH, TA-MG, TA-SOL), and lateral margin of stability and its variability calculated at heel-strike and at the minimum for each stance phase. We first sought to gain insight about acute and time-dependent effects of optical flow perturbations as well as corresponding aftereffects. Here, we performed a one-way, repeated-measures analysis of variance (rmANOVA) on the average of each outcome measure taken across the following 1-min time windows of interest: the last minute of Baseline, the first (“Early”) and last (“Late”) minute of exposure to optical flow perturbations, and the minute following cessation of perturbations (“Post”). When a significant main effect of time was found using an alpha level of 0.05, we performed the following planned, post-hoc, pairwise comparisons: Baseline versus Early, Baseline versus Late, Early versus Late, and Baseline versus Post. Finally, for kinematics-based outcome measures found to have a significant pairwise comparison between Early and Late in the primary rmANOVA, we used a two-way rmANOVA to test for significant main effects and interactions between time (Early versus Late) and experimental session (perturbations versus control). We report effect size using partial eta squared (η_p_^2^) for main effects and Cohen’s d for pairwise comparisons. We excluded a subset of data for one subject (i.e., EMG and MoS) due to EMG saturation and missing kinematic marker data. For all analyses, we defined significance using an alpha level of 0.05 and define differences that yield *p*-values in the range of 0.05 ≤ *p* < 0.07 as statistical trends approaching significance.

## Results

Table 1 summarizes individual subject characteristics. We found no statistically significant difference in baseline kinematic measurements between the experimental and control sessions.

### Initial response

**Fig. 2:**
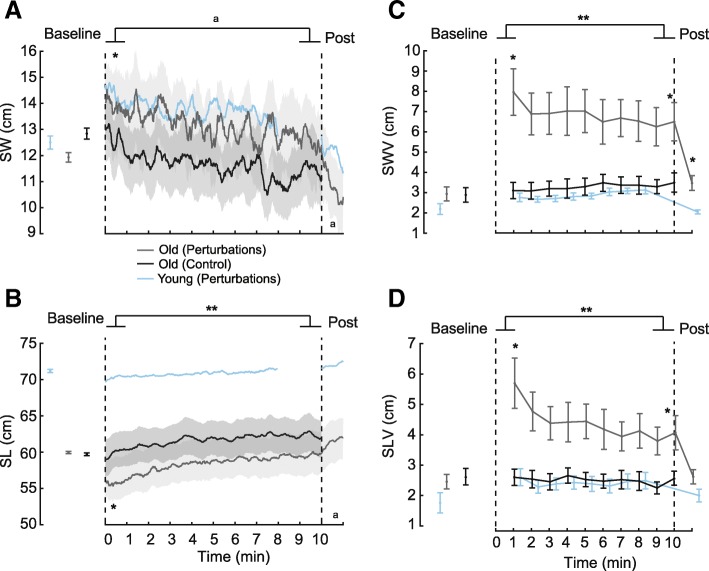
Effects of prolonged exposure to optical flow perturbations on foot placement kinematics. Group average (±standard error) of perturbation-induced effects on **a **step width (SW), **b** step length (SL), **c** step width variability (SWV), and **d** step length variability (SLV) for older participants. The area between the vertical dashed lines represents the period when visual perturbations were present. Previously published reference data from young participants walking for 8 min with optical flow perturbations is shown for comparison. The repeated measures ANOVA revealed significant (*p* < 0.001) main effects across the perturbation session (baseline, early, late, post) for all outcome measures. Single asterisks (*) indicate significantly different from baseline values in older adults (*p* < 0.05). Double asterisks (**) indicate significantly different between early and late time points for prolonged exposure in older adults. We also note differences approaching significance for post and baseline for step width (*p* = 0.056), between early and late for step width (*p* = 0.054), and between post and baseline for step length (*p* = 0.054) in older adult participants (^a^)

**Fig. 3:**
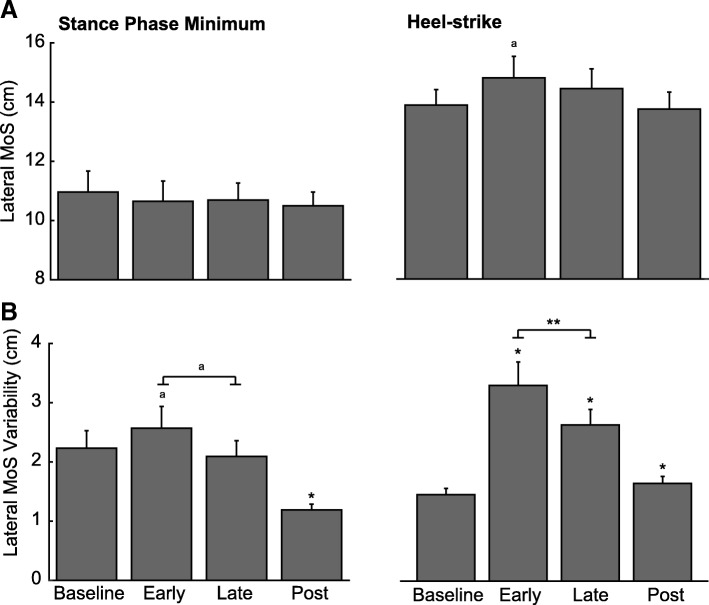
Effects of prolonged exposure to optical flow perturbations on lateral margin of stability (MoS) and its variability in older participants. Group average (±standard error) (**a**) lateral MoS and (**b**) its associated variability calculated at its stance phase minimum and at heel-strike for the perturbation session. The repeated measures ANOVA revealed significant main effects across the perturbation session (baseline, early, late, post) for lateral MoS at heel strike (*p* = 0.012), the variabilities of lateral MoS at its stance phase minimum (*p* < 0.001) and at heel strike (*p* < X). Single asterisks (*) indicate significantly different from baseline values in older adults (*p* < 0.05). Double asterisks (**) indicate significantly different between early and late time points for prolonged exposure in older adults (*p* < 0.05). We also note differences approaching significance between baseline and early for lateral MoS at heel-strike (*p* = 0.051) and between early and late for stance phase minimum lateral MoS variability (*p* = 0.065) (^a^)

**Fig. 4:**
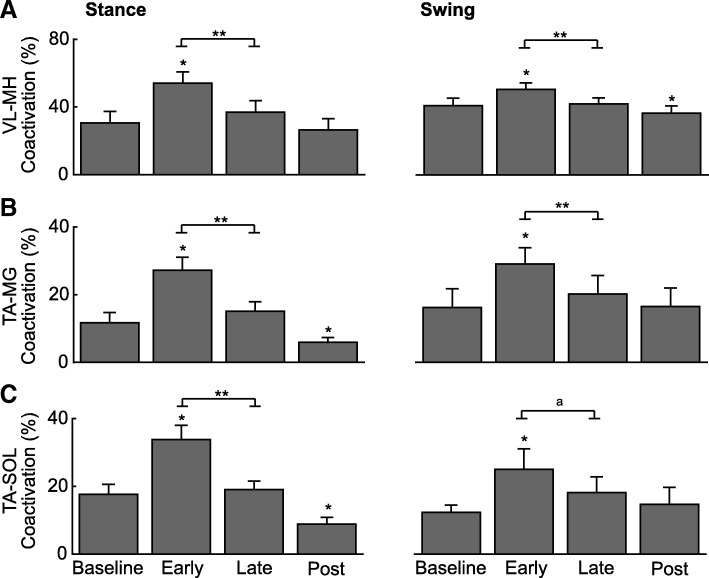
Effects of prolonged exposure to optical flow perturbations on antagonist leg muscle coactivations. Group average (±standard error) percent coactivation between (**a**) vastus lateralis (VL) and medial hamstring (MH), **b** tibialis anterior (TA) and medial gastrocnemius (MG), and **c** tibialis anterior and soleus (SOL) during the stance and swing phases of strides taken during the perturbation session in older adults. The repeated measures ANOVA revealed significant main effects across the perturbation session (baseline, early, late, post) for all outcome measures (*p*-values≤0.020). Single asterisks (*) indicate significantly different from baseline values in older adults (*p* < 0.05). Double asterisks (**) indicate significantly different between early and late time points for prolonged exposure in older adults (*p* < 0.05). We also note differences approaching significance between early and late for TA-SOL coactivation during swing (*p* = 0.066) (^a^)

In the first minute of exposure to optical flow perturbations, older adults took 17% wider (t = 2.66, *p* = 0.021, d = 0.74) and 7% shorter (t = 3.85, *p* = 0.002, d = 1.07) steps and increased their step width and step length variabilities by 171% (t = 5.34, *p* < 0.001, d = 1.48) and 132% (t = 4.68, *p* = 0.001, d = 1.30), respectively, compared to Baseline (Fig. 2). By comparison, in young adults, the same perturbations increased step width and step length by only 14% and 2%, and their variabilities by only 39% and 62%, respectively. For older adults, although the minimum MoS during stance was unaffected by perturbations, MoS at heel-strike tended to increase by 6%, on average, compared to Baseline (t = 2.19, *p* = 0.065, d = 0.63) (Fig. 3a). Step-to-step variability of MoS at heel-strike increased significantly (t = 4.47, *p* = 0.001, d = 1.29) while that of minimum MoS tended to increase (t = 2.15, *p* = 0.055, d = 0.62) following the onset of perturbations (Fig. 3b). Also compared to Baseline, changes in gait kinematics were accompanied by roughly twice the stance and swing phase antagonist coactivation for all three leg muscle pairs (2.13 ≤ t ≤ 5.01, *p*-values≤0.042, 0.62 ≤ d ≤ 1.45) (Fig. 4).

### Time-dependent changes in walking balance control

From early to late exposure, step length increased significantly (t = 2.98, *p* = 0.011, d = 0.83) while step width tended to decrease (t = 2.14, *p* = 0.054, d = 0.59), and both values returned to values seen at Baseline (0.40 < t < 0.87, p-values≥0.403) (Fig. 2a-b). Step width and step length variabilities decreased significantly (t = 2.67, *p* = 0.021, d = 0.74 and t = 2.46, *p* = 0.030, d = 0.68, respectively) but remained elevated compared to Baseline walking (t = 4.95, *p* < 0.001, d = 1.37 and t = 3.31, *p* = 0.006, d = 0.92, respectively) (Fig. 2c-d). Step length and step width exhibited significant main effects of session, with shorter, wider steps in the perturbation session (F_3,36_ = 24.62, *p* < 0.001, η_p_^2^ = 0.672 and F_3,36_ = 16.48, *p* = 0.002, η_p_^2^ = 0.579, respectively) but with no significant time×session interactions. Step length variability showed an effect of session (F_3,36_ = 9.19, *p* = 0.010, η_p_^2^ = 0.434) and time (F_3,36_ = 11.32, *p* = 0.006, η_p_^2^ = 0.486), but no significant interaction. In contrast, step width variability exhibited significant main effects of session (F_3,36_ = 5.09, *p* = 0.043, η_p_^2^ = 0.298) and time (F_3,36_ = 11.06, df = 3, *p* = 0.006, η_p_^2^ = 0.480), and a significant time×session interaction (F_3,36_ = 8.55, *p* = 0.013, η_p_^2^ = 0.416). The interaction revealed that step width variability in older participants modestly increased with time during normal walking but decreased with prolonged exposure to perturbations.

We found no time-dependent effects on minimum MoS, but an effect approaching significance for MoS at heel-strike (F_3,36_ = 4.82, *p* = 0.051, η_p_^2^ = 0.305) (Fig. 3a). Variability of MoS at heel-strike decreased significantly from Early to Late exposure (t = 2.27, *p* = 0.044, d = 0.66), and a similar effect on the variability of minimum MoS approached significance (t = 2.01, *p* = 0.065, d = 0.58). MoS at heel-strike remained elevated compared to Baseline (t = 4.46, *p* = 0.001, d = 1.29), while minimum MoS returned to Baseline values (t = 0.62, *p* = 0.551, d = 0.18) (Fig. 3b). Across experimental sessions, minimum MoS variability and MoS variability at heel-strike exhibited significant main effects of session (F_3,36_ = 20.38, *p* = 0.001, η_p_^2^ = 0.649 and F_3,36_ = 29.52, *p* < 0.001, η_p_^2^ = 0.729, respectively) and time×session interactions (F_3,36_ = 6.42, *p* = 0.028, η_p_^2^ = 0.369 and F_3,36_ = 5.82, *p* = 0.034, η_p_^2^ = 0.346, respectively). The interaction revealed that minimum MoS variability and MoS variability at heel-strike tended to increase with time during normal walking, but decreased with prolonged exposure to perturbations. MoS variability at heel-strike also had a main effect approaching significance for time (F = 4.13, *p* = 0.067, η_p_^2^ = 0.273), tending to decrease with prolonged exposure to perturbations.

Stance and swing phase antagonist muscle coactivation decreased significantly from Early to Late exposure (2.34 ≤ t ≤ 4.91, *p*-values≤0.040, 0.68 ≤ d ≤ 1.42), returning to values seen at Baseline for nearly all of the tested muscles; only differences in swing phase TA-SOL coactivation failed to exceed the criterion for significance (t = 2.04, *p* = 0.066, d = 0.59) (Fig. 4).

### Aftereffects following cessation of optical flow perturbations

In the minute immediately following cessation of optical flow perturbations, participants tended to adopt longer and narrower steps than at Baseline, with effects approaching significance (t = 2.14, *p* = 0.054, d = 0.59 and t = 2.12, *p* = 0.056, d = 0.59, respectively) (Fig. 2). In addition, step width variability averaged 18% larger for Post than Baseline (t = 5.45, *p* < 0.001, d = 1.51) (Fig. 2). Compared to baseline values, these aftereffects were accompanied by significantly less variable minimum MoS (− 43%, t = 3.53, *p* = 0.005, d = 1.02) but modestly higher variability of MoS at heel-strike (+ 13%, t = 3.39, *p* = 0.006, d = 0.98) (Fig. 3b). Finally, older adults walked with significantly less antagonist coactivation following cessation of perturbations compared to Baseline (Fig. 4). Specifically, stance phase TA-MG and TA-SOL coactivation averaged 49% (t = 2.52, *p* = 0.028, d = 0.73) and 50% (t = 3.92, *p* = 0.002, d = 1.13) less, respectively, and swing phase VL-MH coactivation averaged 11% (t = 2.98, *p* = 0.013, d = 0.86) less at Post than at Baseline (Fig. 4).

## Discussion

In a randomized, crossover design that included a control session, we used this study to investigate the propensity for time-dependent tuning of walking balance control and the presence of aftereffects in older adults following prolonged exposure to optical flow balance perturbations. We report data in support of each of our three hypotheses, and discuss below our interpretation that older adults: (*i*) respond to the onset of perturbations using generalized anticipatory balance control, (*ii*) deprioritize that strategy following prolonged exposure to perturbations, and (*iii*) upon removal of perturbations, exhibit short-term aftereffects that indicate a lessening of anticipatory control, an increase in reactive control, and/or an increase in balance confidence.

Consistent with prior studies, older adults were disproportionately susceptible to the onset of optical flow perturbations compared to previously published reference data in young adults [8, 16]. For example, step width and length variabilities, indicators of corrective motor responses to preserve balance, increased by 2 to 3 times more in older adults than in young adults compared to unperturbed walking. In addition, there was support for our hypothesis that older adults would adopt a generalized anticipatory balance control strategy at the onset of perturbations. For instance, during the first minute of exposure, older adults walked with wider and shorter steps, larger MoS at heel-strike, and greater antagonist leg muscle coactivation than during unperturbed walking. Walking with wider steps increases the lateral base of support and shorter steps can better position the body’s CoM within those margins while serving to resist unexpected slips or trips [38]. In addition, antagonist muscle coactivation is often interpreted as a feedforward, anticipatory neural response to balance challenges. Specifically, increased antagonist muscle coactivation likely acts to bolster leg joint stiffness and mitigate the effects of unexpected balance disturbances [19, 21]. Alternatively, or perhaps in some combination, the same response may be attributed to reduced balance confidence at the onset of perturbations; indeed, a fear of recurrent falls has been independently linked to elevated antagonist leg muscle coactivation [20]. Because participants were unaware of the particular timing of the onset of perturbations, the adoption of this generalized anticipatory strategy, seen previously in young participants [23], is a logical response to an unexpected and unfamiliar balance challenge. Simultaneously, high variability in foot placement suggests that this strategy was not entirely successful, still requiring participants to orchestrate adjustments from one step to the next. Although the minimum stance phase MoS was unaffected by perturbations, on average, it too exhibited much larger variability than during unperturbed walking and for some steps even became negative. Greater step-to-step variability in minimum MoS following the onset of perturbations may help explain why participants adopted larger foot placement variability; changes in foot placement can correct deviations in CoM trajectory to preserve walking balance.

With prolonged exposure to perturbations, older adults exhibited time-dependent changes in metrics of walking balance control that we interpret as evidence for an underlying change in balance control strategy. Despite the continued presence of perturbations, step width, step length, MoS at heel-strike, and all antagonist muscle coactivation outcomes returned to values observed during normal, unperturbed walking. Though more modest, we previously observed similar time-dependent changes in step width and length in young participants [23]. One potential interpretation is that participants simply “tuned out” during prolonged exposure, or perhaps down-weighted visual feedback deemed unreliable, and thereby disregarded the destabilizing nature of these perturbations. However, we continue to suspect these explanations are unlikely given that step-to-step *variability* in step width, step length, and margin of stability remained significantly elevated from baseline even after 10 min. Rather, we posit that older adults, like young adults previously, abandoned, or at least deprioritized, generalized anticipatory control in favor of task-specific, reactive control. In this context, corrective motor adjustments, measured in this study as increased movement variability, are orchestrated as participants perceive the instantaneous threat to their balance and plan and execute an appropriate and task-specific reactive response from one step to the next. A similar behavior might also be accompanied, or perhaps explained, by multi-sensory reweighting away from visual feedback and toward an increased awareness of available sensory information [13]. We previously posited that young adults [23] exhibit such a shift away from generalized anticipatory control following prolonged exposure to perturbations due to the high metabolic energy cost associated with walking with shorter, wider steps, and greater antagonist muscle coactivation [5, 39]. A similar explanation may apply here to older participants as well. However, prolonged exposure to perturbations in our older participants may have also promoted an increase in their confidence to successfully accommodate the disturbances without falling, particularly compared to their response to the unexpected onset of those perturbations. In either event, we would interpret these time-dependent effects as favorable outcomes during perturbation training. In addition, based on recently published EEG data [40], the time-dependent changes we report here may be simultaneously accompanied by altered cortical activation; Peterson et al. (2018) found that intermittent optical flow perturbations in young participants walking on a balance beam increased electrocortical activity in the parietal, occipital, and cingulate areas [40]. Those authors interpreted their findings to suggest that such perturbations promoted motor learning of a balance task in brain areas associated with integrating visual information with motor coordination.

Finally, we found short-term aftereffects following cessation of optical flow perturbations that lent support to our third hypothesis. Specifically, after 10-min of walking with perturbations and their subsequent cessation, older adults walked with longer and more narrow steps, moderate increases in foot placement variability, and roughly half the minimum MoS variability and antagonist lower leg muscle coactivation as they did before training. Compared to baseline walking, we would interpret these effects as indicating lessening of anticipatory control, a persistent and disproportionate use of reactive balance control, and/or increased balance confidence in our older adult participants. While speculative, a training environment that offers older adults the opportunity to practice task-specific reactive balance control in the presence of balance challenges during walking, particularly given these after effects, may have functional advantages. During everyday tasks, older adults must navigate complex environments while orchestrating corrections in posture and foot placement, for example obstacle avoidance or in response to a physical balance disturbance. However, many older adults succumb to deficits in motor coordination compared to young adults [41] while facing more dangerous consequences of falling [42]. Moreover, reduced balance confidence and a fear of falling can independently limit community engagement and, thereby, health and independence [43, 44]. Intuitively, older adults likely use generalized anticipatory control during walking, adopting wider and shorter steps and increased antagonist leg muscle coactivation compared to young adults to avoid errors in corrective motor responses that could precipitate a fall. However, those changes can be maladaptive, inhibiting agonist muscle function and the ability to generate rapid, task-specific balance corrections. As a proof of concept, our study builds confidence in larger clinical trials to test the efficacy and translational utility of optical flow perturbation training, including potential effects on responses to unexpected, “real-world” like balance challenges.

One unexpected finding was that, despite an increase in its variability, and unlike MoS at heel-strike, average minimum stance phase MoS was highly resilient in the face of balance perturbations. This is consistent with work from Young and Dingwell [33], who proposed that preserving a certain minimum MoS is an important control goal for maintaining balance in walking. MoS is an aggregate measure, reflecting the interaction between lateral foot placement and mediolateral CoM motion – which increases in response to optical flow perturbations [24]. Together, our findings suggest that step-to-step changes in lateral foot placement may help to regulate the relative position of body CoM and base of support to preserve minimum MoS during the stance phase. Future work should explore the extent to which stance phase minimum MoS is preserved across challenging balance tasks in walking in different populations at risk of falls.

We acknowledge several limitations of our study. First, we opted not to collect EMG data during the control session, presuming that comparisons across days would be unreliable due to differences in sensor placement, orientation, etc. Thus, we cannot exclude the possibility that reduced antagonist coactivation would also present following prolonged unperturbed treadmill walking. We observed time-dependent changes in step width and step length during the control session that resemble those due to prolonged exposure to perturbations, although at smaller magnitudes. Nevertheless, we know of no evidence to date suggesting that higher antagonist coactivation in older adults changes, for example, during treadmill acclimation. We also acknowledge that our VR paradigm is not designed to be ecologically representative of a specific balance challenge faced by older adults during everyday tasks. Nevertheless, we contend that these perturbations need not directly resemble such a challenge to be useful; the neuromuscular responses that they elicit remain meaningful and highly relevant to walking balance integrity. In addition, our experiment was not designed to establish the timeline for washout of aftereffects following perturbation cessation. Instead, we randomized the session order for our subjects to control for any ordering effects that may be expected due perturbation exposure. Finally, our older adult participants were also independent in their community and may not be representative of those most likely to need training to improve walking balance.

## Conclusions

Older adults exhibited time-dependent changes in walking balance control and short-term aftereffects in response to prolonged exposure to optical flow perturbation training. Specifically, older adults appeared to deprioritize general anticipatory control with prolonged exposure to perturbations, evidenced by a return of step width, step length, MoS, and antagonist coactivation to baseline values. Short-term after effects, for example the marked reduction in antagonist coactivation, are indicative of a lessening of anticipatory control, an increase in reactive control, and/or an increase in balance confidence that require further study. We consider this an early, proof-of-concept study into the clinical utility of prolonged exposure to optical flow perturbations as a training tool for corrective motor adjustments relevant to walking balance integrity toward reinforcing task-specific, reactive control and improving balance confidence in older adults.

## Additional files

Additional file 1: Sample video showing mediolateral optical flow perturbations applied in our custom virtual reality environment. (MP4 6433 kb)

Additional file 2: Individual subject data for primary outcome measures. (XLSX 28 kb)

## Data Availability

Data supporting this paper are submitted as electronic supplementary material (Additional file 2) to be made available in the online version of the manuscript.
